# Yield Visualization Based on Farm Work Information Measured by Smart Devices

**DOI:** 10.3390/s18113906

**Published:** 2018-11-13

**Authors:** Yoshiki Hashimoto, Daisaku Arita, Atsushi Shimada, Takashi Yoshinaga, Takashi Okayasu, Hideaki Uchiyama, Rin-Ichiro Taniguchi

**Affiliations:** 1 Faculty of Information Science and Electrical Engineering, Kyushu University, 744 Motooka, Nishi-ku, Fukuoka 819-0395, Japan; hashimoto@limu.ait.kyushu-u.ac.jp (Y.H.); atsushi@ait.kyushu-u.ac.jp (A.S.); uchiyama@limu.ait.kyushu-u.ac.jp (H.U.); rin@kyudai.jp (R.-I.T.); 2 Faculty of Information Systems, University of Nagasaki, 1-1-1, Manabino, Nagayo, Nishisonogi, Nagasaki 851-2195, Japan; 3 Institute of Systems, Information Technologies and Nanotechnologies, 2-1-22 Momochihama, Sawara-ku, Fukuoka 814-0001, Japan; yoshinaga@isit.or.jp; 4 Faculty of Agriculture, Kyushu University, 744 Motooka, Nishi-ku, Fukuoka 819-0395, Japan; okayasu@bpes.kyushu-u.ac.jp

**Keywords:** farm work information, position estimation, action recognition, bluetooth beacon, motion sensor

## Abstract

This paper proposes a new approach to visualizing spatial variation of plant status in a tomato greenhouse based on farm work information operated by laborers. Farm work information consists of a farm laborer’s position and action. A farm laborer’s position is estimated based on radio wave strength measured by using a smartphone carried by the farm laborer and Bluetooth beacons placed in the greenhouse. A farm laborer’s action is recognized based on motion data measured by using smartwatches worn on both wrists of the farm laborer. As experiment, harvesting information operated by one farm laborer in a part of a tomato greenhouse is obtained, and the spatial distribution of yields in the experimental field, called a harvesting map, is visualized. The mean absolute error of the number of harvested tomatoes in each small section of the experimental field is 0.35. An interview with the farm manager shows that the harvesting map is useful for intuitively grasping the states of the greenhouse.

## 1. Introduction

### 1.1. Background and Objective

Farm managers have to make many decisions when they farm. For example, in cultivation planning, they have to decide where they farm, what and when they grow in a greenhouse, etc. In farm work management, they also have to decide how they grow their crops and how many people to hire.

Recently, information on crop growth has been acquired and utilized so that farm managers can make appropriate decisions. Growth of crops is affected by the environment of the farm field where crops are planted, so observation of the environment such as temperature, humidity, and solar radiation is important. Farm managers acquire the environmental information of their greenhouse, and, referring to the information, they decide on their farm work, controlling the greenhouse environment, deciding the time or the amount of watering and fertilizing, etc. It becomes possible to increase the yield by utilizing the data and controlling the environment suitable for plants, and to plan the future cultivation of crops.

Many studies on precision agriculture, which is the farming management concept of measuring, visualizing, and controlling farm fields in order to help farm managers, have been conducted and have obtained great results. Great numbers of environmental sensors have been installed in farm fields, various images captured by satellites and drones are used for visualizing the spatial variation of farm fields, i.e., the photosynthesis capacity of plants and nitrogen level of soils, etc., and autonomous tractors in farm fields are controlled according to the sensed data. Recently, platform hubs such as FOODIE [1] and WAGRI [2] have been proposed for managing various information about agricultural domains through food ones. Such platform hubs will accelerate integration and analysis of agricultural information.

Though the introduction of sensors into greenhouses has increased yields of the crops, there still remain some issues. One is the spatial variation of the amount of crops; i.e., the amount of crops differs by place in the greenhouse, which is well known among farm managers. The major cause of the variation is that the environment of the greenhouse where crops are growing widely differs by place. Although the introduction of sensors has enabled a farm manager to measure the environment of the greenhouse, sensor values obtained by the sensors installed are not representative values of the greenhouse. Moreover, satellites are not able to capture inside greenhouses, and drones are not able to fly inside greenhouses, especially in Japanese greenhouses since they have low ceilings due to typhoons. Thus, the farm manager has to plan the farm work in the greenhouse based on limited information.

There are three approaches to obtain environmental information of a greenhouse. The first is to directly measure the environment of the greenhouse by placing many sensors in every part of the greenhouse or by using a sensor system that moves all around the greenhouse measuring the environment. However, this requires a complicated system with a high cost. The second is to infer indirectly the spatially-fine-grained environment from the observation of growth of crop plants. Analyzing the plant growing state in each place, the farm manager can locally control the farm work, including the environment control, fertilizing, and watering. However, technically, it is not easy to automatically measure plant growth by cameras and other sensors, and such a system will become complicated and expensive. The third is to acquire the growth information indirectly by referring to the actions of farm laborers in a similar way to the second method. Farm work is reflected in the growth of the plants and the environmental states of a farm field, and, consequently, the spatial difference of the environmental states is obtained by recording farm work indirectly. According to these considerations, the third approach was adopted since recent advances in smart sensors are able to make farm work recording systems more precise and easier to use without a high cost.

However, not only is manual recording inaccurate and tiresome or time-consuming, but it is also difficult to record spatially and temporally dense information, even if it is supported by electronic devices. Therefore, this paper proposes a method of automatic recording farm work using inertial measurement unit (IMU) sensors and a smartphone, which are attached and held by a farm laborer. Then, using the recorded information, a harvesting map focusing on the yield is created. The harvesting map is a map visualizing the amount of the yields in small sections into which the whole greenhouse is divided.

This paper presents and discusses our experiment to automatically record, using smart devices, farm work information in a greenhouse for tomato cultivation. In Section 1.2, the related works are discussed. In Section 1.3, our experimental field is introduced. Then, Section 2 describes how the farm laborer’s position information was obtained using a smartphone and beacons. In Section 3, the method to acquire the farm laborer’s action information using IMU sensors attached to both wrists is presented. Section 4 describes how the position and action information were combined and visualized as the harvesting map. Some discussions and conclusions are described in Section 5.

### 1.2. Related Work with Farm Work Information

The simplest method is to record farm work information based on farm laborers’ manual input. A naive method is, of course, that farm laborers record their work, during their work or after, with a notebook and a pen, but, usually, the data are not digitized and not used for sophisticated analysis. Therefore, there are smartphone-based applications [3] substituting the notebook, which can produce digitized data for further analysis. Guan et al. [4] developed a system that requests the farm laborer to input farm work information referring to the location information of the laborer provided by a GPS-equipped mobile phone. However, the overhead of the manual recording is not small, and the dense data cannot be acquired, although farm managers know they are quite valuable. To simplify the recording process, there are approaches to install simple electronic devices in a greenhouse. A typical one is “Priva”, in which terminals are installed at the end of each ridge in the greenhouse, and in which the farm laborer records the farm work by pushing buttons [5].

Another possible approach is to use dedicated agricultural tools, such as a handcart or scissors. By measuring the weight of the handcart or by detecting the sound of the scissors cutting something, some farm work can be detected. However, this method is only applicable to specific tasks and not applicable to many other kinds of farm work.

An intelligent approach which analyzes and records farm work by cameras has been proposed [6]. In the proposed system, four red-blue tapes are attached to a farm laborer. The system estimates the farm laborer’s work (watering, seeding, and harvesting) and direction based on image analysis of red-blue tapes using a hidden Markov model. Though the accuracy of farm work estimation shown in [6] is relatively high, the number of cameras needs to be large when the coverage of an actual farm field becomes large. In addition, image analysis is affected by environmental changes such as the lighting conditions, the background, and the clothes of a farm laborer. To solve these issues, the system tends to require complicated configuration.

Considering the related works mentioned above, we have developed a method of automatic farm work recording using inertial measurement unit (IMU) sensors in smartwatches and a smartphone, which are attached to and held by a farm laborer. Such smart devices are becoming popular and their prices are dropping dramatically. In addition, the data recorded can be easily sent to servers via the smartphone, which enables us to easily construct a system to analyze farm work information at low cost.

### 1.3. Experimental Field and System

**Figure 1:**
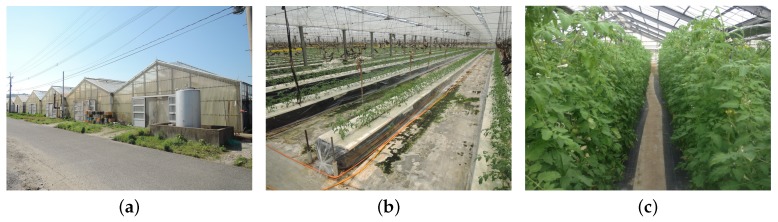
Experimental Field. (**a**) Outside greenhouse; (**b**) inside greenhouse just after planting tomato seedlings; (**c**) inside greenhouse.

Figure 1 shows three photos of the experimental field (a tomato greenhouse). The size of the tomato greenhouse is 45 m long along the ridge and 40 m long perpendicular to the ridge. In the tomato greenhouse, tomato seedlings were planted in August. From September to next June, tomato fruits were harvested on three, four, or five days in a week depending on the season. During the harvesting period, the hight of tomato plants are adjusted to 1.6–1.9 m by farm laborers in order to make daily work easy.

**Figure 2:**
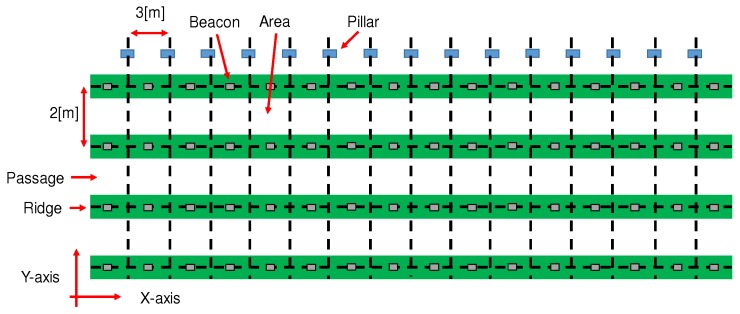
The greenhouse for cultivating tomatoes. There are four ridges and three passages extending across the experimental zone, which is in part of the greenhouse.

The experiment is conducted in the part of the greenhouse, depicted in Figure 2, which includes four ridges separated by three passages, and which is 6 m in width and 45 m in length. The green bold lines represent ridges where tomatoes are planted. There are walking passages between ridges; in these passages, the farm laborer performs the work needed to grow tomatoes. Blue rectangles in the diagram represent pillars supporting the greenhouse. As shown in Figure 2, each passage is divided into 16 sections constituting the units for estimating positions based on the pillars and the ridges. Sixty-four beacons (aplix myBeacon MB004Ac-DR1; the successor model, applix myBeacon MB004Ac-DR2, is JPY25,000 (≒USD220)/10 beacons) are positioned on the ridges in the greenhouse, as shown in Figure 2.

To identify which section the farm laborer works on, in Figure 2, the *X*-axis is set as the axis along the ridges, and the *Y*-axis is set as the axis across the ridges.

This paper defines two ways of specifying the work position of a farm laborer *f*. One way is the use of 2-dimensional coordinate values $p^{f}=\left(p_{x}^{f},p_{y}^{f}\right)$ whose unit is a meter. The other is the use of section-based values $P^{f}=\left(P_{x}^{f},P_{y}^{f}\right)$, indicating a section where a farm laborer *f* is located. In addition, tomato plants on the ridges are too high for farm laborers to jump over, and this causes farm laborers to move only on the passages.

In the experiment, one farm laborer performs harvesting work carrying one smartphone (ASUS Zenfone 2, the price of which was about USD300) and wearing one smartwatch (Sony Smartwatch 3, the price of which was about USD200) on each wrist. The smartphone receives radio waves from beacons placed in the experimental field (details are mentioned in Section 2), and the smartwatches measure arm motion during harvesting work (details are mentioned in Section 3). After harvesting work, the arm motion data are sent from the smartwatches via the smartphone to a cloud system with the history of radio waves in the smartphone. Data analysis are conducted on the cloud system.

Total deployment cost for the experimental field is about USD2100, and that for the whole tomato greenhouse operated by four farm laborers, which is a typical size of a tomato greenhouse in Japan, becomes USD8100.

## 2. Estimation of the Farmer’s Position

### 2.1. Overview

In outdoor farms, GPS seems the most common method for positioning. Large and expensive GPS receivers are attached to tractors. In greenhouses, the estimated position using small and wearable GPS receivers carried by farm laborers contains not only random errors but also systematic errors that are not removable by filtering and are not accurate enough to describe farm work information.

Apart from GPS, there are three approaches to estimate the position of a farm laborer. The first is to estimate an individual’s position using fixed sensor devices such as cameras [7,8,9] and laser range finders (LRFs) [10] in the environment. The advantage of this method is that it yields a relatively accurate position of an individual when the sensors are geometrically calibrated. However, camera-based or LRF-based approaches have to solve the problem of occlusion. The solution usually requires setting a sufficient number of sensors, which leads to complicated and expensive systems.

The second is to estimate the position by Pedestrian Dead Reckoning (PDR), in which wearable devices such as inertial measurement units (IMUs) are used. IMUs, which acquire triaxial acceleration and angular velocity, have been recently embedded in smartphones, and they are becoming quite popular. The advantage of PDR is that there is no limit regarding the measuring area, but it cannot give the absolute position and accumulates estimation errors.

The last method is a combination of mobile devices and fixed devices, such as a smartphone with Wi-Fi access points or Bluetooth beacons [11].

This study has adopted the last approach, i.e., using beacons and a smartphone to estimate farm laborers’ positions, for the following three reasons:

1. Compared with other sensors such as cameras, the maintenance of the beacons is easy, because the battery in each beacon, consisting of two dry cells, lasts for about a year, and the cost of beacons is quite cheap as well.
2. Compared with PDR, which does not require a complicated system, the complexity of the beacon-based system is almost the same, and the accuracy of position estimation is higher because the estimation errors are not accumulated over time.
3. Unlike GPS, the beacons’ radio waves are less affected in a greenhouse, and the result acquired by the beacon waves is more accurate.

This section presents a method to estimate the position of a farm laborer in a greenhouse, which is a very important element of farm work information. In the greenhouse, multiple beacons are placed that broadcast Bluetooth UUID (Universal Unique IDentifier: a 128-bit number used to identify the beacon), and a smartphone is used to receive these signals for position estimation. Within this environment, RSSIs (Received Signal Strength Indicators: a strength of the radio wave received by a device such as smartphone) of the radio signals broadcast by the beacons are measured by the smartphone, and from the RSSIs, the farm laborer’s position in units of “section” described in Section 1.3 is estimated.

### 2.2. Estimation Method

Our method consists of the following three steps. First, the farm laborer’s approximate position is estimated from RSSIs broadcast by beacons. Next, the *X*-position is smoothed with the mode function. The final step is smoothing the *Y*-position using the map matching technique.

**Figure 3:**
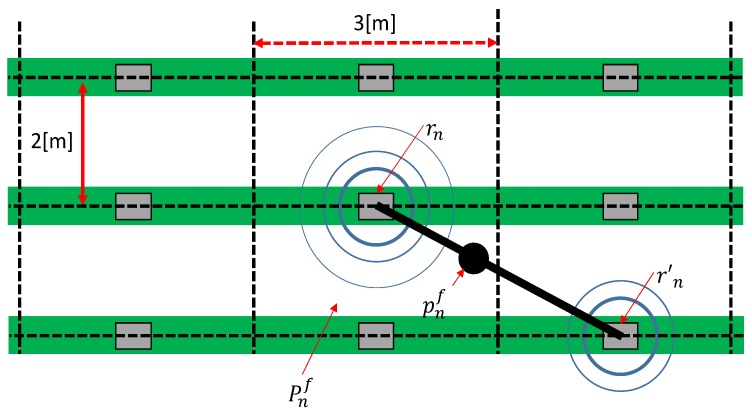
Diagrammatic depiction of the estimation method. First the approximate farm laborer’s position is estimated $p^{f}\left(n\right)$ with $p_{i_{1}}\left(n\right)$ and $p_{i_{2}}\left(n\right)$. Next, $p^{f}\left(n\right)$ is transformed into an section label $P^{f}\left(n\right)$.

Let $r^{f}\left(n\right)=\left\{r_{1}\left(n\right),r_{2}\left(n\right),\cdots ,r_{N_{b}}\left(n\right)\right\}$ be a time series of RSSIs, where *f* is the farm laborer ID, $N_{b}$ represents the total number of beacons, *n* is the sampled discrete time $n=\frac{t}{T^{P}}$, and $T^{P}$ is the sampling interval of the beacon signal reception. When multiple signals from the same beacon *i* are received between the two consecutive sampled times, $r_{i}$ is set to be the average of those. In case no signal is received from the *i*-th beacon during that period, $r_{i}$ is set to 0. At first, the farm laborer’s approximate position is calculated. Suppose the position of the beacon giving the strongest RSSI is $p_{i_{1}}\left(n\right)=\left(p_{i_{1},x}\left(n\right),p_{i_{1},y}\left(n\right)\right)$ and that the second strongest RSSI is $p_{i_{2}}\left(n\right)=\left(p_{i_{2},x}\left(n\right),p_{i_{2},y}\left(n\right)\right)$. The farm laborer’s approximate position is approximately calculated from $p_{i_{1}}\left(n\right)$ and $p_{i_{2}}\left(n\right)$ as $p^{f}\left(n\right)=\left(p_{x}^{f}\left(n\right),p_{y}^{f}\left(n\right)\right)$ using the following equations:

(1) $$p_{x}^{f}\left(n\right)=\frac{\left(1+\epsilon \right)p_{i_{1},x}\left(n\right)+p_{i_{2},x}\left(n\right)}{2+\epsilon }$$

(2) $$p_{y}^{f}\left(n\right)=\frac{\left(1+\epsilon \right)p_{i_{1},y}\left(n\right)+p_{i_{2},y}\left(n\right)}{2+\epsilon }$$

where ϵ is a positive constant that is sufficiently low to avoid placing $p^{f}\left(n\right)$ in a borderline area. The calculated 2-D position $p^{f}\left(n\right)$ is then transformed to section-based description, $P^{f}\left(n\right)=\left(P_{x}^{f}\left(n\right),P_{y}^{f}\left(n\right)\right)$ as shown in Figure 3.

The second step of the estimation process is to smooth or to reduce spike noises in the *X*-position. The smoothed position ${\bar{P}}^{f}\left(n\right)=\left({\bar{P}}^{x}\left(n\right),{\bar{P}}^{y}\left(n\right)\right)$ is calculated using the following equations:

(3) $${\bar{P}}_{x}^{f}\left(n\right)=Mode_{n-W_{x}}^{n+W_{x}}\left(P_{x}^{f}\left(n\right)\right)$$

(4) $${\bar{P}}_{y}^{f}\left(n\right)=Mode_{n-W_{y}}^{n+W_{y}}\left(P_{y}^{f}\left(n\right)\right)$$

where $Mode_{n_{s}}^{n_{e}}\left(L_{n}\right)$ is defined as a function returning most frequently appeared value in $\left\{L_{n}|n_{s}\leq n\leq n_{e}\right\}$. The actual filter sizes are experimentally defined as $W_{x}=3,W_{y}=3$.

**Figure 4:**
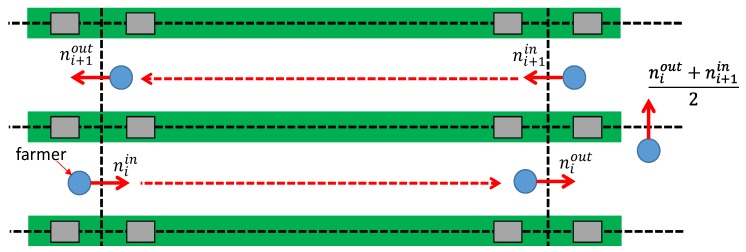
Passage restriction. A farm laborer can move from one passage to the next only by walking to the end of a passage. Thus, the farm laborer must be in the same passage from $n_{i}^{in}$ to $n_{i}^{out}$.

The final step is to reduce the noise in the *Y*-position and to determine which passage the farm laborer is located in. In the greenhouse where tomatoes are cultivated, the height of the plants prevents the farm laborer from jumping over the tomato plants; in other words, the farm laborer is not able to cross over to another passage. Thus, the farm laborer is only able to move from one passage to another by walking around the end of a passage. This movement restriction makes it possible to determine which passage the farm laborer is located in by applying the mode function to the *Y*-position. The final estimation result ${\bar{\bar{P}}}^{f}\left(n\right)=\left({\bar{\bar{P}}}_{x}^{f}\left(n\right),{\bar{\bar{P}}}_{y}^{f}\left(n\right)\right)$ is obtained as follows:

(5) $${\bar{\bar{P}}}_{x}^{f}\left(n\right)={\bar{P}}_{x}^{f}\left(n\right)$$

(6) $${\bar{\bar{P}}}_{y}^{f}\left(n\right)=\left\{\begin{array}{cc} Mode_{n_{i}^{in}}^{n_{i}^{out}}\left({\bar{P}}_{y}^{f}\left(n\right)\right) & \left(n_{i}^{in}\leq n\leq n_{i}^{out}\right)\\ {\bar{\bar{P}}}_{y}^{f}\left(n_{i}^{out}\right) & \left(n_{i}^{out}\leq n\leq \frac{n_{i}^{out}+n_{i+1}^{in}}{2}\right)\\ {\bar{\bar{P}}}_{y}^{f}\left(n_{i+1}^{in}\right) & \left(\frac{n_{i}^{out}+n_{i+1}^{in}}{2}\leq n\leq n_{i+1}^{in}\right) \end{array}\right\}$$

where $n_{i}^{in}$ and $n_{i}^{out}$ denote the beginning and end times, respectively, of the *i*-th instance in which the laborer walks in the middle of the passage, as depicted in Figure 4. The method expressed in Equation (6) estimates in which passage the farm laborer is, applying the mode function from time $n_{i}^{in}$ to $n_{i}^{out}$. At the end section of a passage, where the farm laborer is able to move to another passage, the farm laborer’s position is estimated forcefully based on Equation (6) referring to $n_{i}^{out}$, $(n_{i}^{out}+n_{i+1}^{in})/2$, and $n_{i+1}^{in}$.

### 2.3. Experiments and Discussion

An experiment was conducted in the greenhouse. A farm laborer walked through three passages with a smartphone, held 1 m above the ground, which receives radio waves from beacons. The duration of the experiment was about 25 min. Approximately 120,000 UUIDs were received and 1390 position samples were subsequently estimated.

**Figure 5:**
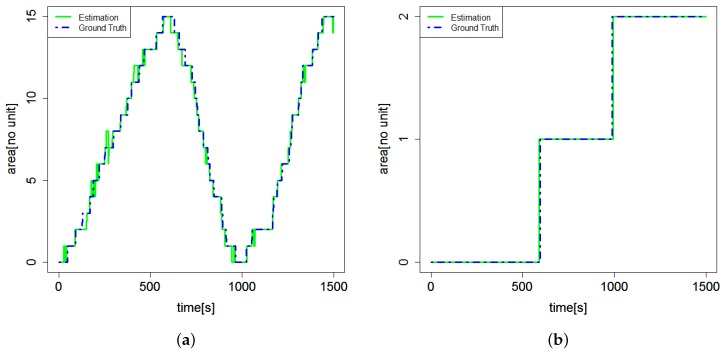
The result of the position estimation. (**a**) shows the *X*-position section label. (**b**) shows the *Y*-position section label.

Figure 5 shows a sample of the results of the position estimation conducted for the farm laborer in this experiment. The solid lines indicate the estimated positions, and the dashed blue lines indicate the manually obtained ground truth. Figure 5a shows that over a period of time the farm laborer moves from Unit Number 0 to Unit Number 15 and back from 15 to 0 as each passage is divided into 16 sections. On the other hand, Figure 5b shows that the farm laborer moves from the 0th passage to the 1st and 2nd passages. By comparing the estimated position with the ground truth at every sampled time *n*, the rates of successfully estimated position samples to the total samples 1390 were 86%, 100%, and 86% along the *X*-position, *Y*-position, and both axes respectively.

The restricted movement of the farm laborer contributed to the achievement of a high degree of accuracy along the *Y*-position. Along the *X*-position, only 86% accuracy was achieved. The reason for lower accuracy is that sometimes the strongest RSSI received by a smartphone was not broadcast by the beacon which was the nearest to the smartphone, though the difference in the signal strengths is very small.

## 3. Farmer’s Action Recognition

### 3.1. Overview

In addition to the farm laborer’s position, the farm laborer’s action, a time sequence of harvesting actions, is necessary for obtaining harvesting work information. Since harvesting work time of each farm laborer is contingent on the farm laborer’s starting and finishing the application that measures the arm motion (instead of this application, other farm information systems such as Priva [5] can be used), the system has to obtain the times at which farm laborers harvest tomatoes one by one from the arm motion data during harvest work.

**Figure 6:**
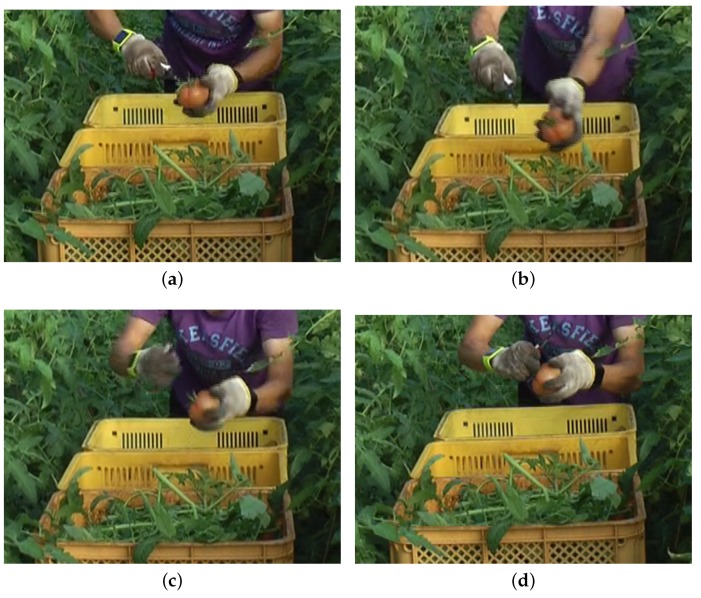
The harvesting action of a farm laborer. A farm laborer holds a tomato in his left hand and is about to cut off the stem of the tomato (**a**). After cutting the stem off, the laborer stretches out both hands to throw the stem into a waste box attached to a cart (**b**,**c**). As a final action, the laborer brings his hands back to the initial position (**d**).

According to our prior observations, farm laborers harvest a tomato in three steps: cutting a tomato from a tomato plant, cutting the stem of the tomato, and putting the tomato into a container. The farm laborer who cuts the stem of a tomato performs a unique arm motion (Figure 6). The motion occurs only when the farm laborer cuts the stem of a tomato, and the motion is distinguished from other motions performed during harvesting work.

In this paper, the action of cutting a stem of a tomato is called the harvesting action, and the system recognizes this motion of the harvesting action from all harvesting work. The unique motion of this harvesting action makes recognition of harvesting action so easy that a simple recognition method is able to be adopted.

**Figure 7:**
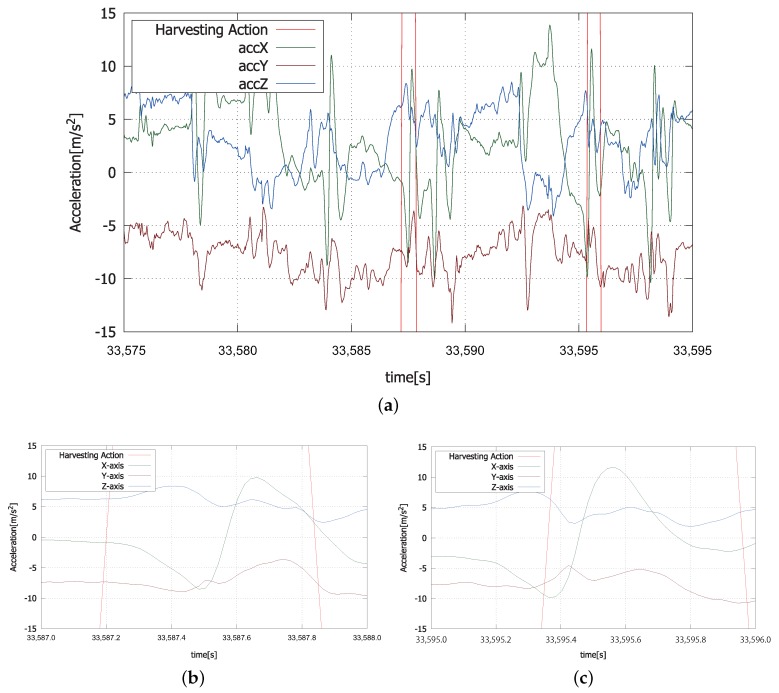
Two examples of acceleration sequence of harvesting action. (**a**) Two examples of harvesting action indicated between red lines; (**b**) enlargement of the first harvesting action in (**a**); (**c**) enlargement of the second harvesting action in (**a**).

When a harvesting action is performed, triaxial acceleration sequences as shown in Figure 7 are acquired from IMUs, or smartwatches, worn by the farm laborer. Figure 7a shows a part of the smoothed triaxial acceleration sequence of the right wrist, which includes two harvesting actions indicated by red lines. Figure 7b,c shows the time-magnified sequences of the first and the second harvesting actions shown in Figure 7a. Acceleration sequences of the harvesting actions are similar to each other. Characteristic acceleration patterns are recognized through machine learning. Random forest has been adopted for this experiment.

### 3.2. Recognition Method

#### 3.2.1. Feature Representation

The system classifies all actions in harvesting work into harvesting action and normal action by acceleration and angular velocities. To classify actions in a harvesting work, first, raw time series data are smoothed because the sensor data include a considerable amount of high-frequency noise in each axis, which hinders high recognition performance. To smooth the raw time series data, the weighted moving average method is applied to each of triaxial accelerations and angular velocities.

**Figure 8:**
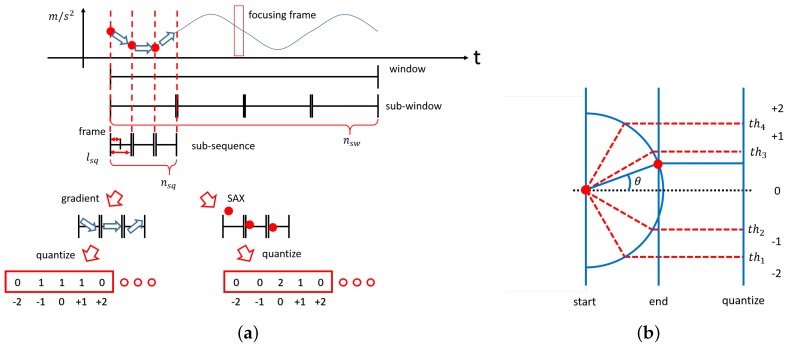
Diagrammatic depiction of the calculation of a feature vector. (**a**) Window for generating histograms; (**b**) gradient features quantized into five levels.

A feature vector represents a series of acceleration data in a fixed window size $l_{w}$, and, as shown in Figure 8a, the window-sized data is divided into multiple sub-windows, each of which is, again, divided into multiple sub-sequences. Here, let $n_{sw}$ be the number of sub-windows in a window, let $n_{sq}$ be the number of sub-sequences in a sub-window, and let $l_{sq}$ be the length of a sub-sequence, or the number of frames in a sub-sequence. Therefore, one window size $l_{w}$ is defined as $l_{w}=n_{sw}\times n_{sq}\times l_{sq}$.

Next, a sequence of acceleration data in a sub-sequence is transformed into a single quantized value. To achieve the quantization, two representations are used: one is Symbolic Aggregate approXimation (SAX) [12], which represents the magnitude of acceleration sequences; the other is the gradient of acceleration [13,14], which represents the amount of change in acceleration sequences. In SAX, a sequence is symbolically represented, and, here, the acceleration sub-sequence is quantized into a single constant value from −2 to +2. The gradient of acceleration is calculated as the angle between the start and end values of the sub-sequence, and it is also quantized into a single constant value from −2 to +2 by simple thresholding shown in Figure 8b. Then, a feature histogram, or a histogram of the quantized data, is calculated in each sub-window as shown in Figure 8a. Finally, the $2\times n_{sq}$ histograms generated in the $n_{sq}$ sub-windows in a window are concatenated to represent a feature vector of the window.

#### 3.2.2. Action Recognition

Our goal is to recognize the harvesting actions from the entire time series data acquired during harvesting work. Therefore, the recognition is a two-class discrimination problem: harvesting actions as a positive class and normal actions as a negative class. In other words, a one-versus-rest strategy is applied [15], and the classification is performed in random forest [16,17], a machine learning method. Here, one window datum is extracted from acceleration sequences as a positive sample representing one harvesting action regardless of the length of the action. The extracted window corresponds in the start time with the positive sample. On the other hand, as negative samples, multiple window data are extracted frame by frame from the sequences representing non-harvesting periods. Random forest is then trained with the feature vectors of those positive samples and negative samples.

In a testing phase, windowed data are extracted, frame by frame, from the entire acceleration sequence of all the farm work, and each of windowed data is represented in a feature vector. When a one-versus-rest strategy is applied, random forest produces a time series of a posteriori probability $\left(0\leq a^{f}\left(m\right)\leq 1,0\leq m<M\right)$ as the output for each class—in this experiment, for the harvesting action or the normal action within each frame, where *m* is the sampled discrete time $m=\frac{t}{A}$, and $T^{A}$ is the sampling interval of the IMU. Harvesting actions are located in the sequence based on the following rules:

1. Representative time of each harvesting action is decided by finding, in the sequence, the local maximum of $a^{f}\left(m\right)$ which is greater than $th_{a}$.
2. If the difference between a representative time and the following timeis smaller than $2\times l_{w}$ frames, the latter is ignored.
3. Based on the local maximum $a^{f}\left(m\right)$, a harvesting action $A^{f}\left(m\right)$ is determined, which indicates that a farm laborer harvests (or does not harvest) a tomato at discrete time *m*.

Consequently, $A^{f}\left(m\right)$ indicates that a farm laborer *f* harvests ($A^{f}\left(m\right)=1$) or does not harvest ($A^{f}\left(m\right)=0$) a tomato at a discrete time *m*.

### 3.3. Results and Discussion

For this experiment, the triaxial acceleration sequences for both wrists of a farm laborer were measured by two smartwatches worn on each wrist of the farm laborer in 50 [Hz] frequency for about 30 min twice on different days. The sequence obtained on the first day was used for training, and the sequence obtained on the second day was used for testing. In this experiment, $n_{sw}=5,n_{sq}=5$, and $l_{sq}=2$ [frame]. The window size $T_{1}$ was 50 [frame] = 1.0 [sec]. For the test phase parameter, $th_{p}=0.5$.

**Figure 9:**
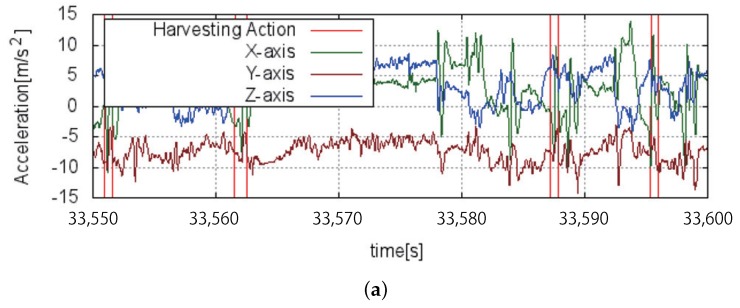

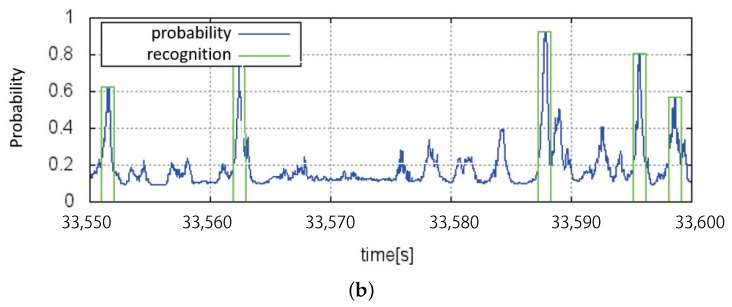
Action recognition output. (**a**) Acceleration sequences and ground truth of harvesting action indicated by red lines; (**b**) probability derived from Random Forest indicated by blue line and recognition results of harvesting action indicated by green lines.

The recognition results are depicted in Figure 9. Triaxial acceleration and the ground truth of a harvesting action are depicted in Figure 9a, and the a posteriori probability output derived from random forest and recognition results are shown in Figure 9b. If the difference between the representative time of a harvesting action recognized indicated by the red line in Figure 9a and a harvesting action in the ground truth indicated by the green line in Figure 9b is small enough, or less than 50 [frame], the harvesting action is considered to be successfully recognized. The precision rate is 93%, which is the rate of the number of successfully recognized harvesting actions to the total number of harvesting actions recognized. The recall rate is 99%, which is the rate of the number of successfully recognized harvesting actions to the number of harvesting actions in the ground truth.

## 4. Harvesting Map

### 4.1. Overview

In the previous sections, methods for measuring the position action information of farm laborers are proposed. Position information is obtained as ${\bar{\bar{P}}}^{f}\left(n\right)=\left({\bar{\bar{P}}}^{x}\left(n\right),{\bar{\bar{P}}}^{y}\left(n\right)\right)$, and this indicates the section in a greenhouse of farm laborer *f* at a discrete time *n*. Action information is obtained as $A^{f}\left(m\right)=\left\{0,1\right\}$, and this indicates that a farm laborer *f* harvests (or does not) a tomato at a discrete time *m*. To generate a harvesting map, the position and action information must be combined, because these two types of information are obtained separately.

First, the discrete time of position and action information is synchronized based on the time of the action information, in order to determine the section ${\bar{\bar{P}}}^{f}\left(m\right)$ where the farm laborer harvests a tomato with the harvesting action at time *m*. Therefore, position information ${\bar{\bar{P}}}^{f}\left(n\right)$ is converted to ${\overset{ˇ}{\bar{\bar{P}}}}^{f}\left(m\right)$, where $n=\lfloor m\frac{T^{A}}{T^{P}}\rfloor$. The harvesting map of farm laborer *f*, $H_{\overset{ˇ}{\bar{\bar{P}}}}^{f}$, is the 2-dimensional histogram of $\left\{\overset{ˇ}{\bar{\bar{P}}}=\overset{ˇ}{\bar{\bar{P}}}{}^{f}\left(m\right)|A^{f}\left(m\right)=1\right\}$, of which each bin $\overset{ˇ}{\bar{\bar{P}}}$ indicates the number of tomatoes harvested in section $\overset{ˇ}{\bar{\bar{P}}}$ by farm laborer *f*. Finally, the harvesting map $H_{\overset{ˇ}{\bar{\bar{P}}}}$ is generated by the equation $H_{\overset{ˇ}{\bar{\bar{P}}}}=\sum_{f}H_{\overset{ˇ}{\bar{\bar{P}}}}^{f}$.

### 4.2. Results and Interview

**Figure 10:**
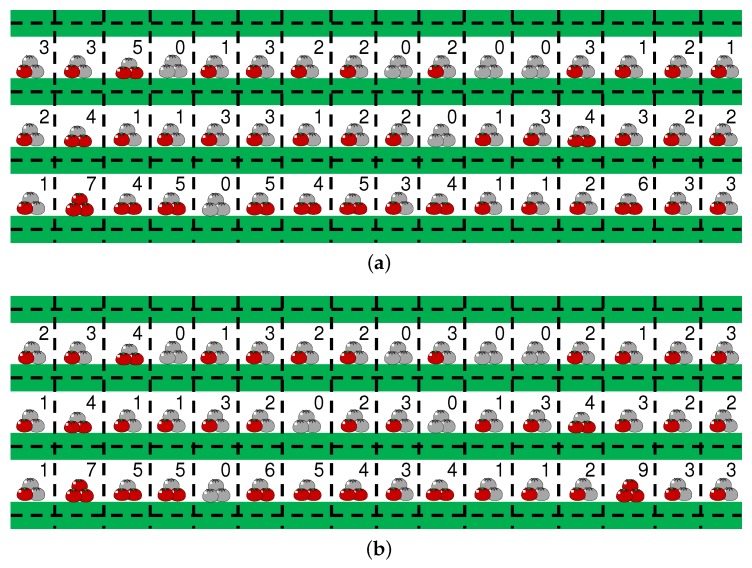
Harvesting maps generated using recognition results (**a**) and ground truth (**b**). As depicted in Figure 2, the green lines in bold line denote ridges and the black dotted lines denote the boundary lines of sections. The number of harvested tomatoes within each section was visualized with a pictogram and real values.

A harvesting map $H_{\overset{ˇ}{\bar{\bar{P}}}}$ is generated as shown in Figure 10a, where a green bold line denotes a ridge, and black dotted lines delineate sections. The image of a tomato within each section represents the percentage to the ideal amount of harvested tomatoes $H_{\overset{ˇ}{\bar{\bar{P}}}}/\tilde{H}$, where $\tilde{H}$ is the ideal amount of harvested tomatoes in a section, and here $\tilde{H}$ is 9. The harvesting map illustrates the spatial variability of yields or the plant states in the greenhouse. Figure 10b illustrates an example of a harvesting map generated from the manually counted ground truth. The mean absolute error of the number of harvested tomatoes in a section was 0.35. Comparing Figure 10a and Figure 10b, the automatically estimated numbers of tomatoes in most sections were close to the ground truth.

To obtain feedback from a farm manager, we interviewed him showing him this harvesting map as depicted in Figure 10b. He is the owner of the greenhouse where our experiments were conducted. He stated that the spatial variation tendency in the harvesting map was quite similar to his estimation of it. In addition, he stated that the harvesting map was easy to understand, because of the division of the farm field into small sections according to the ridges and pillars. Therefore, the detailed description of the state of the yields can be a good tool for the verification of environmental control and farm work management.

## 5. Conclusions

This paper proposes a new approach to visualizing spatial variation in plant status in a tomato greenhouse based on farm work information operated by farm laborers. Farm work information consists of a farm laborer’s position and action. The farm laborer’s position is estimated based on radio wave strength measured using a smartphone carried by the farm laborer and Bluetooth beacons placed in the greenhouse. The farm laborer’s action is recognized based on motion data measured using smartwatches worn on both wrists of the farm laborer. As an experiment, harvesting information operated by one farm laborer in part of a tomato greenhouse was obtained with 86% accuracy of the farm laborer’s position estimation. A 93% precision rate and a 99% recall rate regarding the farm laborer’s action recognition were achieved. Based on the harvesting information, the spatial distribution of yields in the experimental field, called a harvesting map, was visualized. The mean absolute error of the number of harvested tomatoes in each small section of the experimental field is 0.35. An interview with the farm manager shows that the harvesting map is useful for intuitively grasping the states of the greenhouse.

There are mainly three future works: first, for position estimation, the number of beacons can be reduced to minimize cost and maintain position estimation accuracy. Secondly, for action recognition, the proposed method needs to be extended to multiple farm laborers. Finally, for visualization, it is important to generate other kinds of maps that visualize the spatial distribution of various fields throughout the year, mapping such parameters as plant growth and photosynthesis capacity, based on not only harvesting but also other farm work information.
